# Colonic hiatal hernia leading to obstruction after minimally invasive McKeown esophagectomy: a case report

**DOI:** 10.3389/fsurg.2025.1543955

**Published:** 2025-04-10

**Authors:** Danyang Ma, Jiang Wang, Tong Li, Mingyuan Pang, Hengxiao Lu

**Affiliations:** ^1^ Clinical Medical College, Shandong Second Medical University, Weifang, Shandong, China; ^2^ Department of Thoracic Surgery, Weifang People’s Hospital, Weifang, Shandong, China; ^3^ Department of Radiology, Weifang People’s Hospital, Weifang, Shandong, China

**Keywords:** colonic hiatal hernia, McKeown esophagectomy, intestinal obstruction, esophageal squamous-cell carcinoma, esophageal hiatus, post esophagectomy hiatal hiatus

## Abstract

A 62-year-old man presented with acute abdominal pain and signs of bowel obstruction eight months after undergoing a minimally invasive McKeown esophagectomy for esophageal squamous-cell carcinoma. Initial imaging did not reveal a hernia, and conservative management was unsuccessful. Re-evaluation of imaging suggested a hiatal hernia, and thoracoscopic exploration confirmed a large hernia with the transverse colon herniating into the thoracic cavity. Surgical repair involved reduction of the herniated colon and repair of the diaphragmatic hiatus. The patient recovered uneventfully. This case highlights the diagnostic challenges of post-esophagectomy hiatal hernias and the importance of prompt surgical intervention.

## Introduction

Post-esophagectomy hiatal hernia (PEHH) is increasingly recognized as a significant complication following minimally invasive esophagectomy (MIE) (1). Although MIE offers several advantages—such as decreased postoperative morbidity and accelerated recovery compared to open esophagectomy—it often involves widening of the esophageal hiatus, disruption of diaphragmatic integrity, and omission of crural repair. These factors predispose patients to herniation of abdominal contents into the thoracic cavity (2). The clinical importance of PEHH lies in its potential to cause severe complications, including acute intestinal obstruction, bowel ischemia, and the need for emergent surgical intervention. Moreover, diagnosing PEHH is challenging because postoperative anatomical alterations obscure normal imaging landmarks, complicating accurate interpretation—a difficulty consistently noted in the literature (3). Here, we present a case of a patient who developed a large hiatal hernia resulting in intestinal obstruction after a minimally invasive McKeown esophagectomy, highlighting the diagnostic complexities and the critical need for prompt surgical management.

## Case presentation

### Initial presentation and management

A 62-year-old male was diagnosed with esophageal squamous-cell carcinoma one year prior. He had undergone neoadjuvant chemotherapy, followed by minimally invasive McKeown esophagectomy and adjuvant radiotherapy, with an uneventful postoperative recovery.

One year after surgery, he presented with dysphagia, raising concern for tumor recurrence. Computed tomography (CT) demonstrated a left-sided pleural effusion but no significant abdominal findings. Investigations focused on excluding recurrence or metastasis. Thoracentesis was performed, with cytology of the pleural fluid negative for malignant cells. Gastroscopy showed no anastomotic stricture or evidence of recurrence. Following drainage of the pleural effusion, the patient's symptoms resolved, and he was discharged.

### Emergency readmission and diagnostic challenge

Three days post-discharge, the patient presented to the emergency department with acute, intermittent lower abdominal pain and bloating lasting approximately 24 h. He reported no flatus or stool passage but denied nausea, vomiting, fever, or chills. Physical examination revealed abdominal distension and tenderness localized to the lower quadrants, without rebound tenderness or guarding. Bowel sounds were diminished.

**Figure 1:**
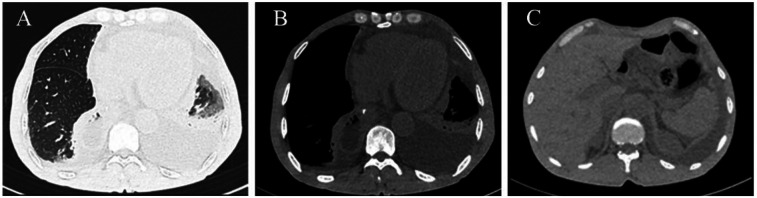
Chest CT findings. **(A,B)** Left medium pleural effusion. **(C)** Significant dilation of the colon.

An enhanced CT scan identified a recurrent left-sided pleural effusion associated with partial collapse of the left lower lung lobe (Figures 1A,B). Notably, marked dilation of the ascending colon and an empty descending colon were observed (Figure 1C). These findings were initially interpreted as an intestinal obstruction. Subtle signs of transverse colon herniation through an enlarged esophageal hiatus were missed, likely due to the obscuring effects of the pleural effusion and the diaphragmatic contour, leading to a delay in accurate diagnosis.

### Clinical deterioration and eventual diagnosis

Despite initial conservative management, the patient's condition worsened over the following three days, presenting with severe abdominal and chest pain, dyspnea accompanied by a sensation of tightness, nausea, vomiting, and abdominal rigidity—raising suspicion for a strangulated intestinal obstruction.

**Figure 2:**
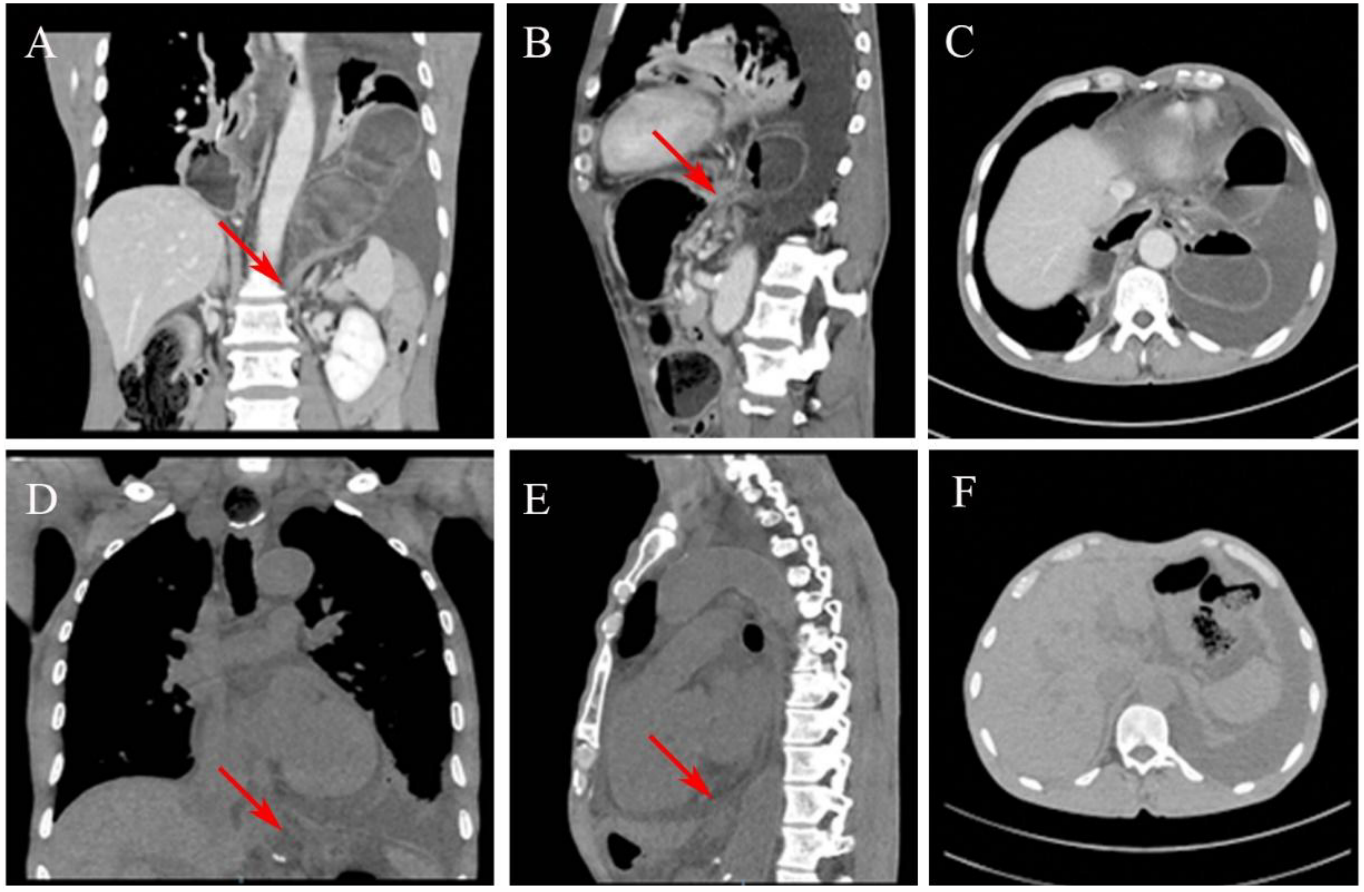
CT images showing the transverse colon herniating through the esophageal hiatus. **(A)** Coronal view and **(B)** sagittal view reveal the herniation into the thoracic cavity. **(C)** The hernia is less distinct on the axial view. **(D,E)** A review of the coronal and sagittal images from the initial chest CT shows a small amount of abdominal fat herniating through the esophageal hiatus into the thoracic cavity. No herniation is visible on the axial view **(F).**

This prompted a detailed reevaluation of his CT scans. Enhanced CT images in the coronal and sagittal planes (Figures 2A,B) clearly revealed herniation of the transverse colon through an enlarged esophageal hiatus into the thoracic cavity. However, the axial view (Figure 2C) was less conclusive due to obscuring effects of the pleural effusion and the diaphragmatic contour, which contributed to the diagnostic difficulty. A review of non-contrast CT scans from the first admission uncovered subtle signs of a small herniation on the coronal and sagittal views (Figures 2D,E). Nonetheless, no evidence of herniation was visible on the axial view (Figure 2F).

### Surgical management and repair

**Figure 3:**
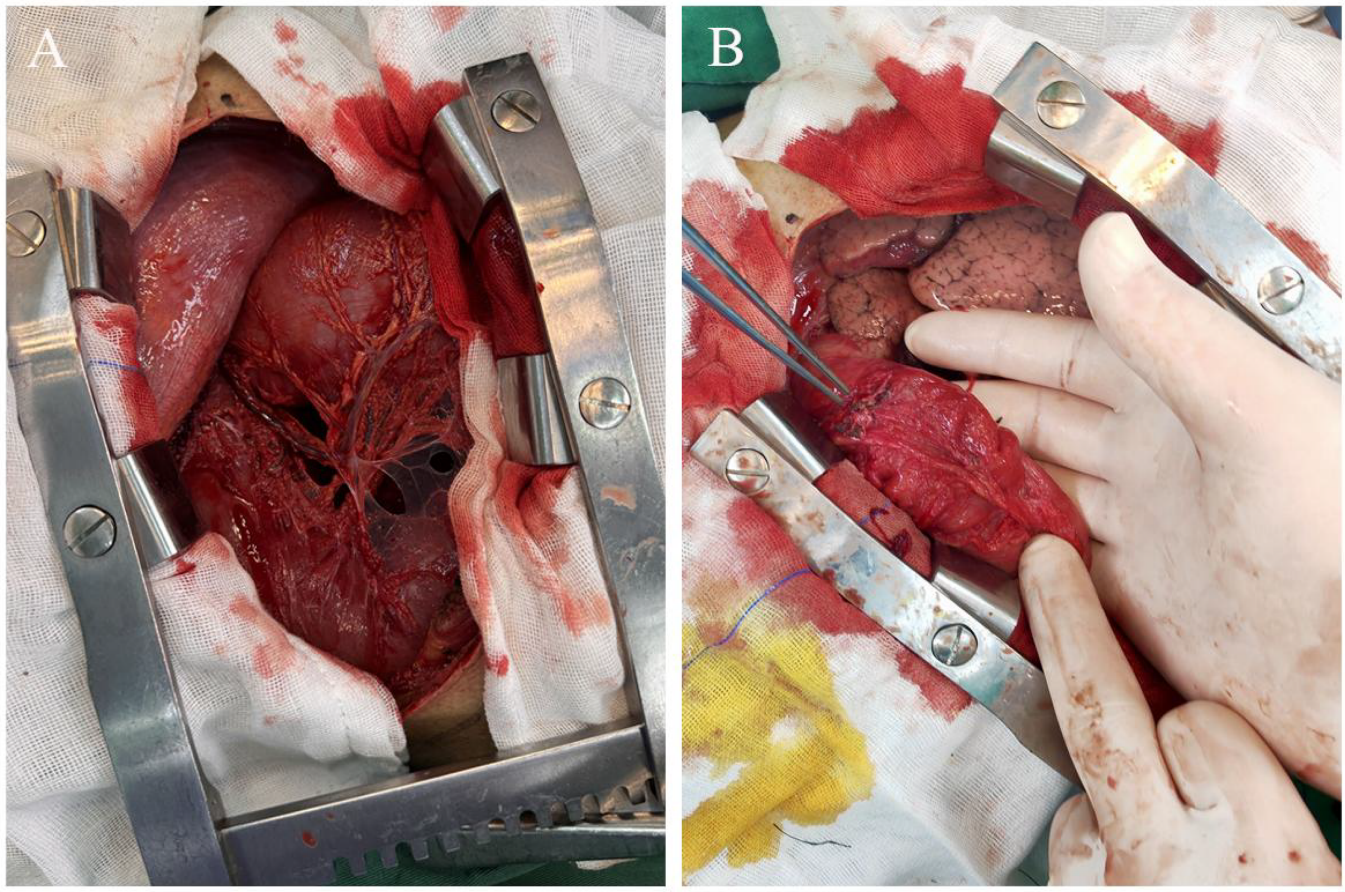
Intraoperative findings. **(A)** A large hiatal hernia containing the transverse colon within the left thoracic cavity. **(B)** The herniated transverse colon is assessed for viability, with no ischemic or necrotic changes noted.

Given the patient's deteriorating clinical condition and concern for a possible strangulated intestinal obstruction, emergency thoracoscopic exploration was performed. Thoracoscopic examination revealed that the transverse colon had herniated into the thoracic cavity. The markedly distended transverse colon and thoracic adhesions impaired visualization and complicated reduction efforts, ultimately necessitating conversion to an open thoracotomy (Figure 3A).

A suction device was used to evacuate the contents of the herniated colon. After decompression, the incision was closed with purse-string sutures, and the colon was assessed for viability, no ischemia or necrosis was observed (Figure 3B). Further inspection confirmed herniation of the transverse colon through an enlarged esophageal hiatus. The colon was reduced into the abdominal cavity, and the hiatus was repaired with nonabsorbable interrupted sutures to prevent recurrence. A chest tube was placed for postoperative drainage.

### Postoperative course

**Figure 4:**
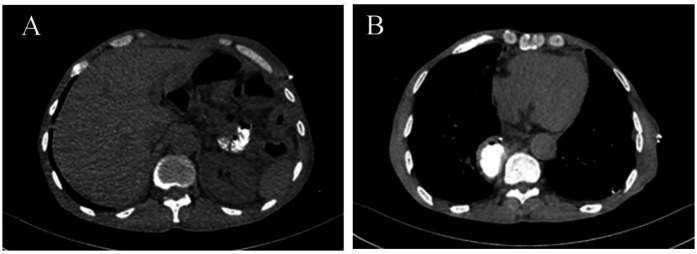
Postoperative imaging. **(A)** Correct positioning of abdominal organs. **(B)** Resolution of the pleural effusion.

The patient's respiratory and gastrointestinal symptoms improved rapidly following surgery. Oral intake was gradually advanced from clear liquids to soft solids without complications. Follow-up imaging demonstrated normal anatomic alignment of the abdominal organs and complete resolution of the pleural effusion (Figures 4A,B). The patient was discharged on postoperative day 7 in stable condition, with plans for routine outpatient follow-up.

## Discussion

### Incidence and changing epidemiology of PEHH

PEHH was a recognized complication even before the widespread adoption of minimally invasive techniques. Traditional open esophagectomy procedures have been associated with relatively low incidence rates, such as 0.69% reported by Price et al. (4) and 0.73% by Gust et al. (5). However, the increasing use of minimally invasive esophagectomy (MIE) appears to be changing this landscape. Studies by Brenkman et al. (6) (MIE: 8.0%, Open: 5.0%), Messenger et al. (7) (MIE: 14.8%, Open: 1.0%), and Lung et al. (8) (MIE: 14.8%, Open or hybrid esophagectomies: 7.5%) highlight the increasing incidence following MIE. Data from Ulloa et al. (9) reported PEHH incidences as high as 8.2% among patients undergoing MIE (Ivor Lewis). Table 1 summarizes detailed incidence data from these key studies. Furthermore, a meta-analysis by Murad et al. (1) revealed an overall PEHH incidence of 3.9% (310 of 7,943 patients), with a significantly elevated risk following MIE (6.5%) relative to open procedures (2.4%), and an odds ratio of 2.76 (95% CI: 1.49–5.11). These observations underscore the evolving epidemiology of PEHH, highlighting its transition from a less frequently reported complication to a recognized long-term postoperative concern, particularly in the context of MIE.

**Table 1:** Incidence and symptomatic rates of post-esophagectomy hiatal hernia (PEHH) across studies.

Author	Esophagectomy procedure	Total patients	Incidence of hernia	Incidence of hernia with different procedure	Symptoms
Price et al. (4)	Ivor Lewis transhiatal substernal interposition	2,182	PEHH: 15 (0.69%)	Ivor Lewis approach: 9/978 (0.92%) Transhiatal approach: 5/601 (0.83%) Substernal interposition:1	Symptomatic: 13 (86.7%) asymptomatic: 2 (13.3%)
Gust et al. (5)	Open laparoscopy	6,608	PEHH: 79 (1.2%)	Open: 0.73% (22/3,010) Laparoscopy: 1.4% (26/1,761)	Symptomatic: 11 (13.9%) asymptomatic: 68 (86.1%)
Brenkman et al. (6)	MIE open	657	PEHH: 45 (6.8%)	MIE: 8.0% (33/432) Open: 5.0% (12/225)	Symptomatic: 31 (68.9%) asymptomatic: 14 (31.1%)
Messenger et al. (7)	MIE open	273	PEHH: 11 (4.0%)	MIE: 9 (13.2%) 0pen: 2 (1.0%)	NA
Lung et al. (8)	MIE open or hybrid esophagectomies	391	PEHH: 36 (9.2%)	MIE: 14.8% (20/135) Open or hybrid esophagectomies: 7.5%(16/212)	Symptomatic: 13 (36%) asymptomatic: 23 (64%)
Hertault et al. (10)	Abdominal phase open abdominal phase minimally invasive	719	Five-year PEHH incidence: 10.3%	Abdominal phase open: 4.5% (13/289) Abdominal phase minimally invasive:10.7% (46/430)	Symptomatic: 27 (45.8%) asymptomatic: 32 (54.2%)
Ulloa Severino et al. (9)	MIE (Ivor Lewis)	390	PEHH: 32 (8.2%)	NA	Symptomatic: 22 (68.8%) asymptomatic: 10 (31.2%)
Lubbers et al. (11)	MIE	307	Incidence of acute PEHH: 8 (2.6%)	NA	NA

PEHH, post-esophagectomy hiatal hernia; MIE, minimally invasive esophagectomy; NA, not available.

### Risk factors and prevent strategies

Risk factors for post-esophagectomy hiatal hernia (PEHH) are multifactorial. Surgical factors have been identified as major contributors; Andreou et al. (12) and Argenti et al. (13) highlighted the widening of the diaphragmatic hiatus during esophagectomy and the lack of crural repair as critical factors in the development of PEHH. Patient related factors also play a significant role. Jain et al. (14) and Iwasaki et al. (15) demonstrated that patients with a low body mass index (BMI <25) and those who have undergone neoadjuvant therapy are at increased risk for PEHH.

Current preventive strategies emphasize meticulous surgical technique, particularly careful diaphragmatic dissection aimed at preserving the structural integrity of the hiatus. Routine assessment of the hiatus after esophagectomy and prompt repair using non-absorbable sutures if defects are identified is recommended. Techniques like cruroplasty and gastropexy may reduce PEHH incidence, but further research is needed to confirm their effectiveness (1, 16).

### Clinical presentation and diagnostic challenges

The clinical presentation of PEHH is highly variable and nonspecific, often complicating diagnosis. While many patients are initially asymptomatic, some develop symptoms over time—such as chest or abdominal pain, nausea, vomiting, dysphagia, reflux, dyspnea, or bowel obstruction. Symptomatic rates, as shown in Table 1, reflect this heterogeneity, ranging widely: Gust et al. (13.9%, 11/79) (5), Lung et al. (36.0%, 13/36) (8), Hertault et al. (45.8%, 27/59) (10), Ulloa et al. (68.8%, 22/32) (9), Brenkman et al. (68.9%, 31/45) (6), and Price et al. (86.7%, 13/15) (4). This variability, influenced by patient factors, surgical techniques, and follow-up protocols, underscores PEHH's subtle and diverse nature, emphasizing the need for clinical vigilance and routine post-esophagectomy monitoring.

Diagnosing PEHH can be challenging, as illustrated in this case, where initial imaging failed to detect the hernia, particularly when relying only on standard axial CT views. Previous studies, such as those by Erkmen et al. (17) and Ganeshan et al. (18) have emphasized the importance of carefully reviewing coronal and sagittal reconstructions. These studies found that multi-planar imaging significantly improves the detection of subtle hernias that might otherwise be missed, highlighting the need for a more comprehensive imaging approach in post-esophagectomy patients.

### Surgical management of PEHH: approaches and considerations

Surgical intervention primarily targets symptomatic cases, particularly when complicated by obstruction, incarceration, or strangulation (19). The proportion of patients presenting with symptoms ranges from 68.8% [Ulloa et al. (9)] to 100% [Crespin et al. (20), Messenger et al. (7), Lubbers et al. (11)], although the higher rates originate from smaller cohorts. The choice between minimally invasive repair (laparoscopic or thoracoscopic) and open surgery (laparotomy or thoracotomy) depends on patient stability, hernia extent, and surgeon expertise (21, 22). As summarized in Table 2, reported rates of minimally invasive repair vary widely, from 24.4% [Gust et al. (5)] to 63.6% [Messenger et al. (7)]. In the present case, worsening symptoms and pleural effusion prompted a thoracoscopic approach, revealing a distended transverse colon within the thoracic cavity. Poor visualization necessitated conversion to thoracotomy and intestinal decompression to safely reduce the colon into the abdomen.

**Table 2:** Summary of symptomatic rates, surgical approaches, recurrence, and outcomes in post-esophagectomy hiatal hernia (PEHH) repair across studies.

Author	Total	Symptomatic *n* (%)	Asymptomatic *n* (%)	MIS repair *n* (%)	Open repair *n* (%)	Overall recurrence *n* (%)	Outcome
Hertault et al. (10)	33	27 (81.8%)	6 (18.2%)	20 (60.6%)	13 (39.4%)	10 (30.3%)	Morbidity: 4 (12.1%)
Brenkman et al. (6)	26	25 (96.2%)	1 (3.8%)	9 (34.6%)	17 (65.4%)	4 (15.4%)	Morbidity: 3 (11.5%)
Gust et al. (5)	78	67 (85.9%)	11 (14.1%)	19 (24.4%)	59 (75.6%)	8 (10.3%)	Morbidity: 36 (46%) Mortality: 1 (1.2%)
Crespin et al. (20)	7	7 (100.0%)	0 (0.0%)	3 (42.9%)	4 (57.1%)	1 (14.3%)	NA
Messenger et al. (7)	11	11 (100.0%)	0 (0.0%)	7 (63.6%)	4 (36.4%)	2 (18.2%)	NA
Lubbers et al. (11)	8	8 (100.0%)	0 (0.0%)	4 (50.0%)	4 (50.0%)	3 (37.5%)	Morbidity: 5 (63%)
Lung et al. (8)	14	11 (78.6%)	3 (21.4%)	5 (35.7%)	9 (64.3%)	4 (28.6%)	Mortality: 1 (7.1%)
Ulloa Severino et al. (9)	32	22 (68.8%)	10 (31.3%)	19 (59.4%)	13 (40.6%)	6 (18.8%)	Morbidity: 3 (9.3%)

MIS, minimally invasive surgery.

### Outcomes, morbidity, and recurrence rates in PEHH repair

Outcomes following PEHH repair vary depending on the timing of intervention and the severity of the hernia. Early surgical intervention typically yields favorable results, whereas delayed diagnosis—particularly in cases complicated by strangulation—significantly increases morbidity and mortality. As detailed in Table 2, overall recurrence rates vary considerably, ranging from 10.3% [Gust et al. (5)] to 37.5% [Lubbers et al. (11)]. Postoperative morbidity also demonstrates substantial heterogeneity, with reported morbidity rates ranging from 9.3% [Ulloa et al. (9)] to as high as 63% [Lubbers et al. (11)]. These variations probably result from differing patient characteristics, surgical methods, surgeon expertise, and morbidity definitions. Standardizing surgical practice and postoperative care, alongside prospective studies, would help minimize complications and improve patient outcomes after PEHH repair.

## Conclusion

In conclusion, this case underscores the critical need for early detection and prompt diagnosis of hiatal hernias following esophagectomy, urging clinicians to remain highly vigilant in patients with risk factors such as low BMI, previous neoadjuvant therapy and MIE. The variable and nonspecific presentation of PEHH complicates diagnosis, requiring CT imaging with multiplanar reconstruction—particularly coronal and sagittal views—for precise identification. Additionally, prospective studies to assess the long-term outcomes of various repair strategies and the establishment of clinical guidelines for the management of PEHH are warranted to advance evidence-based practice and optimize patient outcomes.
